# Impact of hydronephrosis grade on surgical parameters and stone-free rates in prone percutaneous nephrolithotomy: a retrospective multivariable study

**DOI:** 10.1515/med-2026-1506

**Published:** 2026-08-14

**Authors:** Adem Tunçekin, Ali Nihat Gökcan, Erkan Arslan, Yasin Aktaş, Hakan Türk

**Affiliations:** Department of Urology, Uşak Training and Research Hospital, Uşak, Türkiye; Department of Urology, Uşak University Faculty of Medicine, Uşak, Türkiye; Department of Urology, Necmettin Erbakan University Meram Faculty of Medicine, Konya, Türkiye

**Keywords:** hydronephrosis, percutaneous nephrolithotomy, kidney calculi, surgical outcomes, complications

## Abstract

**Objectives:**

To assess the effect of hydronephrosis (HN) grade on operative parameters, complication rates, and stone-free rates (SFR) in patients undergoing prone percutaneous nephrolithotomy (PCNL) performed under a standardized surgical protocol.

**Methods:**

This retrospective study included patients who underwent prone PCNL between January 2021 and June 2024. Patients with bilateral stones or anatomically abnormal kidneys were excluded. HN grade was determined by ultrasonography and confirmed with non-contrast computed tomography. Operative parameters, including operative time, number of accesses, hemoglobin drop, transfusion requirement, hospital stay, and complications, were compared among mild, moderate and severe HN groups.

**Results:**

A total of 327 patients were retrospectively screened. After applying the exclusion criteria, 242 patients who underwent prone PCNL between January 2021 and June 2024 were included in the final analysis. The mean age was 51.4 ± 13.9 years. HN was mild in 40.5 %, moderate in 37.6 %, and severe in 21.9 % of patients. Operation time increased significantly with HN grade (p=0.001), while other parameters including SFR (p=0.521) and complication rate (p=0.760) showed no significant differences. Operation time was the only independent predictor of stone-free status (p<0.001). Subgroup analysis according to median stone burden demonstrated that the association between HN grade and operative time persisted only in patients with lower stone burden (≤5.69 cm^3^, p=0.010), whereas this relationship was not significant in patients with higher stone burden (>5.69 cm^3^, p=0.052).

**Conclusions:**

HN grade does not significantly affect surgical success or complication rates after prone PCNL, although higher grades were associated with longer operative times. HN grade alone should not be considered a limiting factor for achieving complete stone clearance. However, stone burden may be a more important determinant of operative complexity in patients with higher stone burden.

## Introduction

Renal stone disease continues to represent a major global health issue, with a rising prevalence driven by metabolic, dietary, and environmental factors [1]. Kidney stone formation is a multifactorial process involving metabolic, anatomical, and inflammatory mechanisms. Inflammatory processes may contribute to stone formation and progression by altering urinary mineral solubility, promoting crystal aggregation, and affecting the renal tubular microenvironment [2], 3]. For patients with large or complex calculi, percutaneous nephrolithotomy (PCNL) remains the standard of care, offering the highest stone-free rates (SFR) among minimally invasive procedures [4]. Despite its well-established efficacy, several anatomical and procedural factors can influence surgical outcomes including stone complexity, access tract selection, and renal morphology [5]. Hydronephrosis (HN) is one of these key variables, as it can alter collecting system anatomy and thereby affect access tract creation, irrigation dynamics, and stone retrieval efficiency during PCNL [6].

Nevertheless, the relationship between HN grade and PCNL outcomes remains unclear. While some studies have indicated that the absence or mild degree of HN may be associated with increased bleeding risk during PCNL, others have suggested that severe HN could influence operative difficulty and overall surgical efficiency [7], 8]. Given these inconsistencies, further clarification is warranted. Such variations among studies may arise from differences in sample size, imaging modalities used for HN classification, and surgeon experience during PCNL procedures [9], [10], [11]. A comprehensive evaluation based on standardized definitions and consistent operative techniques could therefore provide a clearer understanding of the true impact of HN on PCNL performance.

Therefore, the present study aimed to assess the influence of HN grade on operative parameters, complication rates, and SFR in patients undergoing prone PCNL performed under a standardized protocol.

## Materials and methods

### Study design and patient selection

This retrospective study included patients who underwent prone PCNL between January 2021 and June 2024. The study was approved by the Uşak University Faculty of Medicine Ethics Committee (Approval No: 406-406-04, dated 10.07.2024) and was conducted in accordance with the Declaration of Helsinki (as revised in 2013). Informed consent was not applicable due to the retrospective study design. Patients aged ≥18 years with renal stones requiring PCNL and complete perioperative and postoperative follow-up data were included. All patients underwent routine preoperative anesthetic and surgical evaluation before surgery. Patients with uncontrolled coagulopathy or active medical problems were medically optimized before surgery.

Patients who underwent simultaneous bilateral PCNL or staged procedures for the same renal unit were excluded. Simultaneous bilateral surgery was defined as bilateral PCNL performed during the same anesthetic session, whereas staged procedures referred to planned multi-session procedures. Secondary procedures for residual stones were excluded from the primary outcome analysis. Patients with anatomical anomalies, incomplete follow-up, or missing laboratory data were also excluded.

All patients underwent preoperative non-contrast computed tomography (NCCT) for stone evaluation and grading of HN. A sterile urine culture, defined as the absence of bacterial growth, obtained within 7 days before surgery was confirmed in all patients. Preoperative pyuria status was also recorded. Pyuria was defined as >5 leukocytes per high-power field on microscopic urinalysis.

### Surgical technique

Under general anesthesia, all prone PCNL procedures were performed by experienced urologists. A 6 Fr ureteral catheter and an 18 Fr Foley catheter were placed initially. After prone positioning, calyceal puncture was performed under fluoroscopic guidance, followed by guidewire placement and tract dilation using Amplatz dilators up to 28–30 Fr. Stone fragmentation was performed using pneumatic or ultrasonic lithotripsy depending on surgeon preference and stone characteristics. Complete clearance of fragments was confirmed both endoscopically and fluoroscopically. At the end of the procedure, a 16 Fr nephrostomy tube was routinely inserted. Operative time was defined as the time from cystoscopy and ureteral catheter placement to completion of nephrostomy insertion.

### Data collection and definitions

Demographic and clinical data including age, sex, side of surgery, prior urological surgery, stone location (renal pelvis, calyces, or pelvicalyceal system), and grade of HN were retrospectively collected from patient records. Stone size was assessed on preoperative NCCT. Stone burden was calculated using the ellipsoid formula (length × width × height × π/6) and expressed in cm^3^. Preoperative HN grading was assessed using ultrasonography and/or NCCT findings according to the Society for Fetal Urology (SFU) grading system, and patients were categorized based on the degree of pelvicalyceal dilatation. Mild HN was defined as minimal pelvicalyceal dilatation, moderate HN as calyceal dilatation, and severe HN marked pelvicalyceal dilatation with cortical thinning. Imaging assessments were performed as part of routine clinical evaluation by experienced radiologists and urologists. Because of the retrospective study design, blinded reassessment and formal interobserver agreement analysis were not performed.

Operative variables such as operation time, number of access tracts, hemoglobin drop (preoperative minus postoperative day 1 value), transfusion requirement, and hospital stay duration were also recorded. Stone-free status was defined as the absence of residual fragments >2 mm on imaging obtained approximately 1 month after the procedure using NCCT, kidney-ureter-bladder radiography (KUB) or ultrasonography (USG), as clinically indicated. Imaging assessments were performed by experienced urologists and radiologists. Postoperative complications occurring during hospitalization and within 30 days after surgery were recorded and classified according to the Clavien–Dindo grading system.

### Statistical analysis

All statistical analyses were performed using IBM SPSS Statistics for Windows, version 23.0 (IBM Corp., Armonk, NY, USA). The normality of continuous variables was assessed using the Shapiro–Wilk test. Normally distributed variables were expressed as mean ± standard deviation (SD), while non-normally distributed variables were presented as median and interquartile range (IQR). Categorical variables were reported as frequencies and percentages. Comparisons between more than two groups were performed using the Kruskal–Wallis test for non-normally distributed continuous variables. For pairwise post-hoc comparisons, the Mann–Whitney U test with Bonferroni correction was applied. The Chi-square test was used for comparing categorical variables.

Additional subgroup analyses were performed according to median stone burden (≤5.69 cm^3^ vs. >5.69 cm^3^) to evaluate the association between HN grade and operative time after stratification by stone burden. To determine independent predictors of stone-free status, univariable logistic regression analyses were performed for clinically relevant variables. Variables with a p-value <0.1 in univariable analysis were subsequently included in the multivariable logistic regression model. Results were reported as odds ratios (ORs) with 95 % confidence intervals (CIs). Multicollinearity among covariates was assessed using variance inflation factor (VIF) analysis before multivariable regression, and no significant multicollinearity was detected. A p-value of <0.05 was considered statistically significant.

## Results

### Baseline characteristics of patients undergoing prone percutaneous nephrolithotomy

**Figure 1:**
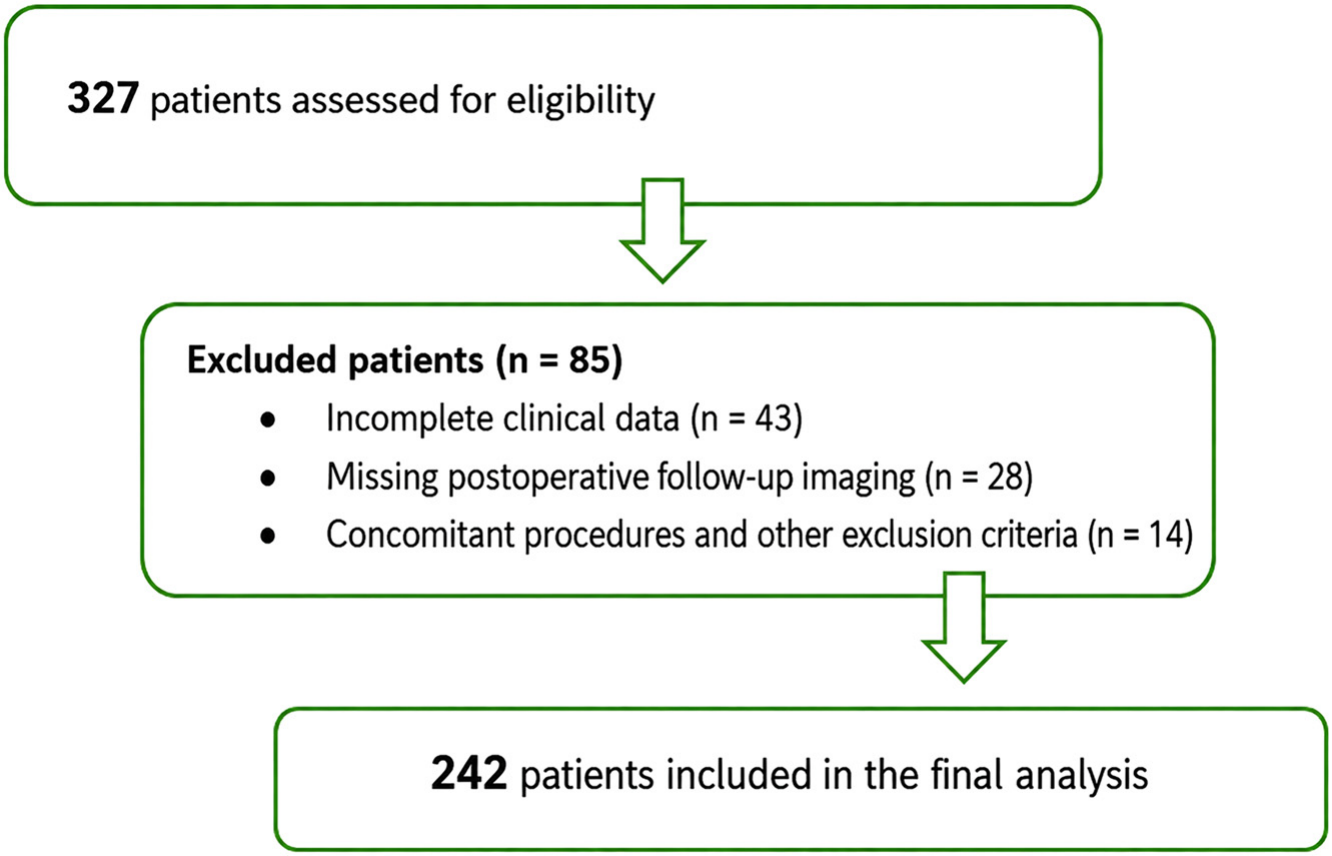
Flowchart of patient selection and study inclusion.

A total of 327 patients were initially screened, and 242 patients met the inclusion criteria and were included in the final analysis. The patient selection process is summarized in Figure 1. The mean age was 51.4 ± 13.9 years, and 144 patients (59.5 %) were male. The procedure was performed on the right side in 109 patients (45.0 %). Stones were most frequently located in the pelvicalyceal system (44.2 %), followed by the calyceal system (31.4 %) and renal pelvis (24.4 %). Stone count distribution (single vs. multiple stones) was comparable among the HN groups (p=0.666). HN grade was mild in 98 (40.5 %), moderate in 91 (37.6 %), and severe in 53 (21.9 %) patients. Stone burden, age, gender, side, stone location, and Hounsfield Unit values were comparable among the groups. Detailed baseline demographic and clinical characteristics stratified according to hydronephrosis grade are presented in Table 1

**Table 1:** Baseline demographic and clinical characteristics stratified according to hydronephrosis grade.

Variable	Mild (n=98)	Moderate (n=91)	Severe (n=53)	p-Value
Age (years)	51.8 ± 13.6	51.3 ± 15.0	51.2 ± 12.7	0.959
Gender				0.209
Male	62 (63.3 %)	56 (61.5 %)	26 (49.1 %)	
Female	36 (36.7 %)	35 (38.5 %)	27 (50.9 %)	
Side				0.766
Right	42 (42.9 %)	41 (45.1 %)	26 (49.1 %)	
Left	56 (57.1 %)	50 (54.9 %)	27 (50.9 %)	
Stone location				0.823
Renal pelvis	24 (24.5 %)	25 (27.5 %)	10 (18.9 %)	
Calyceal	30 (30.6 %)	27 (29.7 %)	19 (35.8 %)	
Pelvicalyceal	44 (44.9 %)	39 (42.9 %)	24 (45.3 %)	
Stone count				0.666
Single	51 (52.6 %)	51 (56.7 %)	26 (49.1 %)	
Multiple	46 (47.4 %)	39 (43.3 %)	27 (50.9 %)	
Stone burden, cm^3^	6.3 (3.3–10.3)	5.9 (2.8–10.3)	4.7 (2.8–7.9)	0.224
Hounsfield unit (HU)	1,084.3 ± 352.2	1,159.1 ± 1,063.1	1,083.9 ± 360.7	0.730

Continuous variables are presented as mean ± standard deviation or median (interquartile range), according to data distribution. Categorical variables are expressed as number (percentage).

### The impact of hydronephrosis grade on perioperative and clinical outcomes

When evaluating the influence of HN grade on perioperative and clinical parameters, a statistically significant difference was observed only in operation time (p=0.001). No significant differences were observed among the groups regarding preoperative pyuria, access count, hospital stay duration, hemoglobin drop, stone-free status, complication status or blood transfusion requirement (all p>0.05). A sensitivity analysis restricted to patients evaluated with NCCT demonstrated findings consistent with the primary analysis, with no significant association between HN grade and stone-free status (NCCT subgroup: p=0.499; overall cohort: p=0.521).

Postoperative complications occurred in 32 (13.2 %) patients, predominantly Clavien–Dindo Grade I–II events, while one patient required angioembolization for postoperative bleeding (Grade III). No complication-related readmissions were observed during follow-up. Detailed perioperative and clinical outcomes are summarized in Table 2. Multivariable logistic regression analysis further demonstrated that HN severity was not an independent predictor of blood transfusion requirement after adjustment for stone burden and operative time.

**Table 2:** Comparison of perioperative and clinical outcomes according to hydronephrosis grade.

Variable	Mild (n=98)	Moderate (n=91)	Severe (n=53)	p-Value
Operation time, min	85 (60–115)	110 (77–135)	120 (75–147)	**0.001**
Hospital stay (days)	1 (1–2)	1 (1–2)	1 (1–2)	0.558
Access count	1 (1–1)	1 (1–1)	1 (1–1)	0.197
Hemoglobin drop, g/dL	1.7 (1.0–2.6)	1.8 (1.2–2.8)	1.6 (1.2–2.2)	0.600
Pyuria	21 (21.4 %)	15 (16.5 %)	4 (7.5 %)	0.091
SFR, n (%)	78 (79.6 %)	66 (72.5 %)	40 (75.5 %)	0.521
Complications, n (%)	15 (15.3 %)	12 (13.2 %)	5 (9.4 %)	0.760
Blood transfusion, n (%)	5 (5.1 %)	14 (15.4 %)	7 (13.2 %)	0.060

Values are presented as median (interquartile range) or number (%), as appropriate. Bold p values indicate statistical significance (p<0.05). SFR, stone-free rate.

Pairwise comparisons using the Mann–Whitney U test revealed that operation time was significantly longer in patients with moderate HN compared to mild HN (p=0.001; 95 % CI for median: 100–120 min vs. 75–100 min), and in those with severe HN compared to mild HN (p=0.003; 95 % CI for median: 95–125 min vs. 75–100 min). However, the difference between moderate and severe groups was not statistically significant (p=0.768). Because operative time was the only perioperative parameter significantly associated with HN grade, additional subgroup analyses stratified by median stone burden were performed to further evaluate this relationship.

Subgroup analysis according to median stone burden demonstrated that the association between HN grade and operative time remained significant in patients with lower stone burden (≤5.69 cm^3^, p=0.010), whereas no significant association was observed in patients with higher stone burden (>5.69 cm^3^, p=0.052). The subgroup analysis results stratified by median stone burden are summarized in Table 3.

**Table 3:** Subgroup analysis of operative time according to hydronephrosis grade stratified by median stone burden.

Stone burden subgroup	Mild	Moderate	Severe	p-Value
≤5.69 cm^3^	80 (60–112.5)	115 (80–135)	120 (90–147.5)	**0.010**
>5.69 cm^3^	90 (62–115)	110 (75–130)	107.5 (70–135)	0.052

Values are presented as median (interquartile range- IQR). Bold p values indicate statistical significance (p<0.05).

### Predictors of stone-free status: univariable and multivariable logistic regression analysis

Univariable logistic regression analyses demonstrated that only operation time was significantly associated with stone-free status (OR=1.014, 95 % CI: 1.008–1.021, p<0.001), whereas HN grade (p=0.499), stone burden (p=0.309), access count (p=0.682), and HU (p=0.668) were not significant predictors. In multivariable logistic regression analysis, operation time remained the only independent predictor of stone-free status (OR=1.019, 95 % CI: 1.011–1.026, p<0.001). HN grade, stone burden, access count, and HU were not independently associated with stone-free status. The univariable and multivariable logistic regression analysis results are summarized in Table 4.

**Table 4:** Univariable and multivariable logistic regression analysis for predictors of stone-free status.

Variable	Univariable OR (95 % CI)	p-Value	Multivariable OR (95 % CI)	p-Value
HN grade	1.342 (0.572–3.150)	0.499	0.965 (0.385–2.420)	0.939
Stone burden	1.028 (0.975–1.085)	0.309	1.025 (0.973–1.079)	0.354
Access count	0.827 (0.333–2.051)	0.682	1.134 (0.420–3.061)	0.804
Operation time	1.014 (1.008–1.021)	**<0.001**	1.019 (1.011–1.026)	**<0.001**
HU	1.000 (1.000–1.001)	0.668	1.000 (0.999–1.001)	0.640

Bold p values indicate statistical significance (p<0.05). Mild HN was used as the reference category. HN, hydronephrosis; CI, confidence interval; HU, hounsfield unit.

## Discussion

In this study, HN grade did not significantly affect the SFR or postoperative complications following prone PCNL. However, operative time increased with higher HN grades, suggesting that anatomical dilation may prolong intraoperative maneuvers without affecting safety or efficiency. Additional subgroup analysis according to median stone burden demonstrated that the association between HN grade and operative time was significant only in patients with lower stone burden, whereas this relationship was not observed in patients with higher stone burden. Although severe HN can create technical challenges during tract creation or stone retrieval, overall outcomes were comparable across HN groups. Similarly, Yucel et al. reported no significant association between HN grade and perioperative parameters after fluoroscopy-guided PCNL, confirming that dilation does not compromise success [12]. Napitupulu et al. also found that patients with severe HN had slightly longer procedures but similar SFR and complication rates compared with mild or moderate HN [13]. Overall, these data support that HN grade alone should not limit PCNL indications when standardized techniques are used by experienced surgeons.

Operative time increased with higher HN grades, likely reflecting technical challenges from altered pelvicalyceal anatomy and limited instrument maneuverability. Subgroup analysis according to median stone burden further demonstrated that the association between HN grade and operative time persisted only in patients with lower stone burden, whereas this relationship was attenuated in patients with higher stone burden. This finding suggests that stone burden itself may become the dominant determinant of procedural complexity in patients with larger stones. These observations are consistent with previous reports demonstrating that anatomical complexity and multi-tract requirements may prolong operative duration without negatively affecting surgical success or complication rates [14]. Similarly, Naeem et al. found that PCNL achieved higher SFR than RIRS, though with longer procedures [15]. Shubham et al. also noted that procedural efficiency in mini-PCNL depends on the lithotripsy device, with Thulium fiber lasers offering shorter times and better fragmentation than conventional systems [16]. Overall, HN related anatomic dilation may extend certain technical steps, but standardized PCNL principles preserve safety and effectiveness. Nevertheless, the relationship between operative time and stone-free status should be interpreted cautiously. Operative duration may also reflect procedural complexity, stone characteristics, and intraoperative decision-making. Therefore, operative time is better considered a process-related indicator rather than a true preoperative predictor of surgical success. Residual confounding and reverse causation cannot be excluded because of the retrospective nature of the study.

Although the difference did not reach statistical significance, the numerically higher transfusion rate observed in the moderate HN group may still be clinically noteworthy. Alterations in collecting system anatomy and renal parenchymal tension associated with hydronephrosis could potentially influence puncture-related bleeding risk during tract creation. Interestingly, Dong et al. reported that absent or mild hydronephrosis may be associated with increased bleeding risk during PCNL, suggesting that the relationship between hydronephrosis severity and hemorrhagic complications may be complex and multifactorial [7]. In the present study, operation time emerged as the only independent predictor of stone-free status. However, this association should be interpreted cautiously, as prolonged operative duration may primarily reflect increased procedural complexity and attempts to achieve more complete stone clearance rather than reduced surgical efficiency itself. Therefore, operative time may function as a surrogate marker of intraoperative complexity in retrospective analyses.

In multivariable analysis, operation time remained the only independent predictor of SFR, whereas HN grade was not independently associated with postoperative success. These findings further support that stone burden, stone complexity, and intraoperative factors may play a more important role in determining surgical outcomes than hydronephrosis severity alone. This finding agrees with previous studies showing that stone burden, location, and complexity rather than HN determine postoperative success [17], 18]. Guy’s Stone Score and S.T.O.N.E. nephrolithometry have been shown to accurately predict SFR, with HN contributing minimally to model performance. Fu et al. also reported that even in the absence of HN, tubeless PCNL achieved similar SFR and complication rates compared with conventional procedures [19]. Overall, intraoperative technique, stone characteristics, and surgeon experience remain the main factors influencing outcomes, not the degree of HN.

The overall complication rate was low, with most events being minor (Clavien–Dindo Grade I–II) such as transient fever or hematuria, and no major complications occurred. These results align with recent studies showing similarly favorable safety profiles for PCNL. Ali et al. reported that most postoperative events were minor and self-limiting, supporting the procedure’s safety even in complex cases [20]. Singh et al. also noted that bleeding and fever were the most common complications but rarely required intervention, confirming that PCNL is safe across diverse patient groups [21]. Ayranci et al. demonstrated that nephrolithometric scoring systems CROES, Guy’s, and S.T.O.N.E. effectively predict complication risk, while HN grade was not an independent predictor of morbidity [22]. Collectively, these findings support that HN severity alone does not substantially influence postoperative safety outcomes in standardized PCNL practice.

This study has several limitations. Its retrospective single-center design may have introduced selection bias, and patient distribution across HN grades was uneven. Although all procedures followed a standardized prone PCNL protocol, minor variations in perioperative management and surgical technique may still have influenced outcomes. HN grading was primarily based on USG and confirmed with NCCT, supporting imaging consistency in routine clinical practice. Previous studies have also emphasized the importance of optimized imaging guidance, standardized dilation techniques, and careful patient selection in minimizing anatomical variability during PCNL [23], [24], [25]. However, postoperative imaging modalities for stone-free assessment were not fully standardized, potentially introducing information bias. Detailed data regarding antibiotic protocols, anticoagulant use, renal insufficiency, tract size, lithotripsy modality, prior ureteral stenting, and tract-related technical variations were not consistently available because of the retrospective design. Finally, blinded reassessment was not performed. Despite these limitations, our findings support that HN grade alone is not a major determinant of surgical difficulty, stone-free status, or postoperative morbidity following PCNL.

Clinically, our findings suggest that HN grade alone should not discourage surgeons from pursuing complete stone clearance during PCNL when appropriate access planning and imaging guidance are applied. Although severe HN may increase technical complexity during renal access or intrarenal navigation, standardized PCNL protocols can maintain comparable surgical success and safety outcomes across different HN grades. The present findings may contribute to improved preoperative risk stratification and operative planning in patients undergoing PCNL. Future prospective multicenter studies integrating anatomical complexity, stone burden, and individualized imaging-based planning are warranted to further clarify the determinants of operative difficulty and surgical success.

## Conclusions

This study showed that HN grade did not significantly affect SFR or postoperative complications in patients undergoing prone PCNL. Although higher HN grades were associated with longer operative times, surgical safety and efficacy remained consistent when standardized techniques were applied. Therefore, HN grade alone should not discourage surgeons from pursuing complete stone clearance during PCNL. However, subgroup analysis suggested that stone burden may be a more important determinant of operative complexity in patients with higher stone burden. Future prospective multicenter studies integrating advanced imaging and individualized surgical planning are warranted to further validate these findings and optimize patient-specific treatment strategies.
